# Quadrotor Identification through the Cooperative Particle Swarm Optimization-Cuckoo Search Approach

**DOI:** 10.1155/2019/8925165

**Published:** 2019-07-24

**Authors:** Nada El gmili, Mostafa Mjahed, Abdeljalil El kari, Hassan Ayad

**Affiliations:** ^1^Applied Physics Department, Cadi Ayyad University, Marrakesh 40000, Morocco; ^2^Mathematics Department, Royal School of Aeronautics, Marrakesh 40000, Morocco

## Abstract

This paper explores the model parameters estimation of a quadrotor UAV by exploiting the cooperative particle swarm optimization-cuckoo search (PSO-CS). The PSO-CS regulates the convergence velocity benefiting from the capabilities of social thinking and local search in PSO and CS. To evaluate the efficiency of the proposed methods, it is regarded as important to apply these approaches for identifying the autonomous complex and nonlinear dynamics of the quadrotor. After defining the quadrotor dynamic modelling using Newton–Euler formalism, the quadrotor model's parameters are extracted by using intelligent PSO, CS, PSO-CS, and the statistical least squares (LS) methods. Finally, simulation results prove that PSO and PSO-CS are more efficient in optimal tuning of parameters values for the quadrotor identification.

## 1. Introduction

Over the past few years, the unmanned aerial vehicles (UAVs) demand has increased dramatically because of the wide range of civilian and military applications. Some of those applications include low cost filming, panoramic picturing, area mapping, surveillance, air pollution monitoring, hostile zones intervention, transmission lines and power distribution inspections, earth science research assistance, etc [1].

In this paper, we are particularly interested in the behavior of one kind of UAVs that has two pairs of rotating rotors attached to the end of a cross named quadrotor or quadcopter. These aircraft are the most complex flying machines due to many physical effects influencing their dynamics including aerodynamic effects, gravity, gyroscopic effects, friction, and inertia. However, they have advantages over conventional helicopters. Given that two motors (the left one and the right one) rotate clockwise and the two others rotate counter clockwise, gyroscopic effects and aerodynamic torques tend to cancel in trimmed flight.

The quadrotor modelling is regarded as a delicate task either using Newton–Euler [2] or Euler–Lagrange [3] formalisms. The model obtained using these approaches is strongly nonlinear, fully coupled, underactuated, and dynamically unstable with complex behavior. So, it is of our interest to explore an efficient model parameter estimation technique to realize precise modelling results without using complicated model structures.

By using systems identification, the quadrotor can be represented by an estimated mathematical model, based only on input and output data considering the aircraft as a black-box process. Therefore, the model's parameter values need to be estimated optimally. In the literature, some classical methods allow the process identification based on the step response such as the Strejc and Broida methods that require aperiodic systems. The integrating process allows identifying systems whose output response corresponds in steady state to a variation in a ramp. However, it is hard to model the unstable divergent behavior of the highly nonlinear quadrotor system using these classical techniques. Other methods raise the problem of identification in statistical terms of parameters estimation. The least squares (LS) method minimizes the squared error between the values predicted by the model and the observed values. This method demonstrated its superiority in parametric identification. In [4], it is shown that the problem of parametric identification of a wiener system could be reduced to a linear parametric estimation problem by a simple input-output data recorded using recursive least squares method (RLS).

The optimization methods of evolutionary algorithms (EAs) and swarm intelligence (SI) techniques can effectively resolve complex optimization problems compared to the described classical and statistical methods. EAs use the survival principle on a set of potential solutions to produce gradual approximations to the optimum, where SI is based on the study of group behavior in decentralized and self-organized societies. Particle swarm optimization (PSO) algorithm [5, 6] and genetic algorithm (GA) [7–9] seem to be the most successful types of EAs and SI, respectively. PSO deals with problems in which a best solution can be represented as a point or surface in a D-dimensional space. This intelligent method has shown superior performances. First, it can escape from local optimization problems. Second, it has no evolution operators such as mutation. Other advantages of PSO method are its less computational complexity compared to GA and its ease of implementation.

Cuckoo search (CS) algorithm is a novel SI algorithm motivated by the aggressive breeding of a bird called “cuckoo.” An advantage of CS compared to PSO and GA is that it uses less number of parameters to be tuned, which makes it more adaptable [10]. Also, the immigration and environmental specifications of cuckoos' groups help to converge and reach best places for breeding and egg laying [10]. Many applications of PSO and CS are proposed in recent works of real discrete optimization problems [11–14], in identification problems [15–18], and for quadrotor's control [19–22]. However, the CS algorithm suffers from its low convergence speed, since it uses a fixed step size over generations. Our proposed cooperative PSO-CS algorithm in [22] combines the social and local search capabilities of PSO and CS. The PSO-CS offers great guidance for cuckoos to the global best positions and ensures a balance between exploitation and exploration of the search space [22].

In this paper, we propose the intelligent PSO and CS methods, the cooperative PSO-CS, and the statistical LS to optimally identify the quadrotor's dynamics under determined operating conditions. The model used to estimate the variations of roll (*ϕ*), pitch (*θ*), yaw (*ψ*) angles, and altitude *z* during the flying process is composed of four subsystems of second order with the same structure and whose coefficients are adjusted with PSO, CS, PSO-CS, and LS to represent as best as possible the quadrotor divergent unstable behaviors. A comparative study is done to highlight the efficiency of the proposed intelligent methods in quadrotor identification.

This paper is organized as follows. In Section 2, Newton–Euler formalism is used to derive the motion equations of the quadrotor. In Section 3, the identification strategy is explained. In Sections 4 and 5, the quadrotor identification is detailed using the proposed intelligent PSO and CS techniques, the cooperative PSO-CS, and the statistical LS. In section 6, the simulation results are given. In the last section, our conclusions are provided.

## 2. Quadrotor Dynamic Modelling

**Figure 1:**
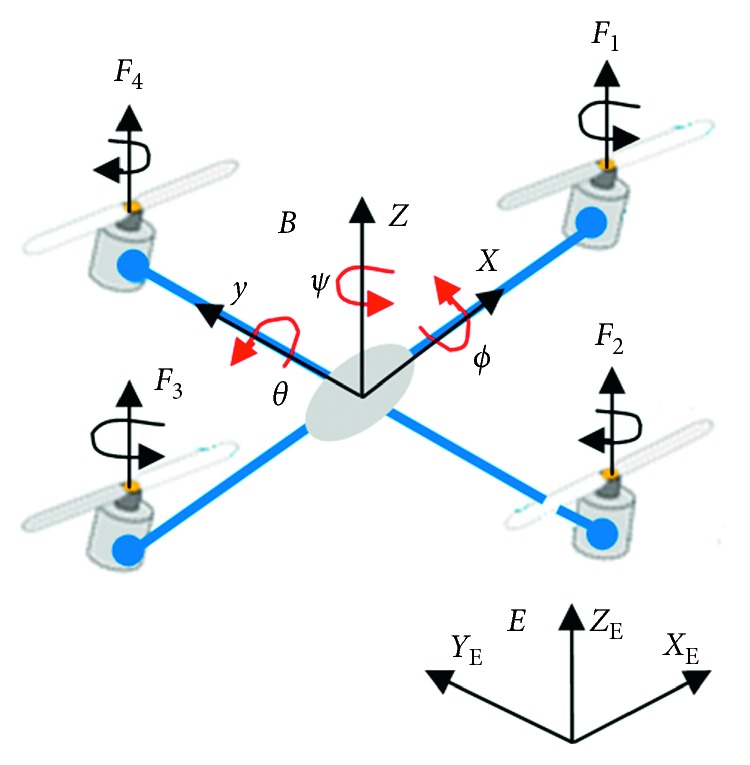
Quadrotor configuration.

The quadrotor is a complex flying machine, which is strongly nonlinear, fully coupled, and underactuated (6-DOF and only four control inputs). Therefore, its aerodynamic is affected by many physical effects, including gravity, gyroscopic effects, friction, and moment of inertia. As shown in Figure 1, the quadrotor consists of two pairs of rotating rotors attached to the end of a cross, and the control electronics is situated in the center of the cross. The two pairs of propellers must spin in opposite directions to prevent the vehicle's overturning. In Figure 1, the absolute position of the mass center is described by the three coordinates (*x*, *y*, *z*) and its attitude by the three Euler's angles (*ϕ*, *θ*, *ψ*).

For simplifying the delicate dynamic modelling of the quadrotor, various working hypotheses have been assumed [1]: the quadrotor structure is assumed rigid and symmetrical (diagonal matrix of inertia); the propellers are assumed rigid (negligible effect of deformation during rotation); a very close approximation of the aerodynamic behavior is assumed (the lift and the drag forces are proportional to the square of the rotational speed of the rotors); and the origin of reference related to the structure is fixed on the center of mass of the quadrotor.

By using Newton–Euler formalism, the equations can be written as follows:

(1) $$\begin{array}{c} \left\{\begin{array}{c} \zeta =v,\\ m\ddot{\zeta }=F_{f}+F_{t}+F_{g},\\ J\dot{\Omega }=M_{f}-M_{a}-M_{gh}-M_{gm},\\ \dot{R}=R\;S\left(\Omega \right), \end{array}\right. \end{array}$$

where *v* is the linear velocity and *m* is the total mass of the quadrotor.

*R* is the rotation matrix:

(2) $$\begin{array}{c} R=\left[\begin{array}{ccc} \cos\;\psi \;\cos\;\theta & \sin\;\phi \;\sin\;\theta \;\cos\;\psi -sin\psi \cos\;\phi & \cos\;\phi \;\sin\;\theta \;\cos\;\psi +\sin\;\psi \;\sin\;\phi \\ \sin\;\psi \;\cos\;\theta & \sin\;\phi \;\sin\;\theta \;\sin\;\psi +\cos\;\theta \;\cos\;\psi & \cos\;\phi \;\sin\;\theta \;\sin\;\psi -\sin\;\phi \;\cos\;\psi \\ -\sin\;\theta & \sin\;\phi \;\cos\;\theta & \cos\;\phi \;\cos\;\theta \end{array}\right]. \end{array}$$

Ω is the angular velocity expressed in the fixed reference:

(3) $$\begin{array}{c} \Omega =\left[\begin{array}{c} \Omega_{1}\\ \Omega_{2}\\ \Omega_{3} \end{array}\right]=\left[\begin{array}{ccc} 1 & 0 & -\sin\;\theta \\ 0 & \cos\;\phi & \sin\;\phi \;\cos\;\theta \\ 0 & -\sin\;\phi & \cos\;\phi \;\cos\;\theta \end{array}\right]\left[\begin{array}{c} \dot{\phi }\\ \dot{\theta }\\ \dot{\psi } \end{array}\right]=W\dot{\eta }. \end{array}$$

*S*(Ω) denotes the oblique symmetric matrix:

(4) $$\begin{array}{c} S\left(\Omega \right)=\left[\begin{array}{ccc} 0 & -\Omega_{3} & \Omega_{2}\\ \Omega_{3} & 0 & -\Omega_{1}\\ -\Omega_{2} & \Omega_{1} & 0 \end{array}\right]. \end{array}$$

*J* is the inertia of the system:

(5) $$\begin{array}{c} J=\left[\begin{array}{ccc} I_{x} & 0 & 0\\ 0 & I_{y} & 0\\ 0 & 0 & I_{z} \end{array}\right]. \end{array}$$

*F*_*f*_ is the total thrust force generated by the four rotors:

(6) $$\begin{array}{c} F_{f}=R\times {\left[\begin{array}{ccc} 0 & 0 & \overset{4}{\underset{i=1}{\sum }}F_{i} \end{array}\right]}^{E}=\left[\begin{array}{c} \cos\;\phi \;\sin\;\theta \;\cos\;\psi +\sin\;\phi \;\sin\;\psi \\ \cos\;\phi \;sin\;\theta \;\sin\;\psi -\sin\;\phi \;\cos\;\psi \\ \cos\;\phi \;\cos\;\theta \end{array}\right]\overset{4}{\underset{i=1}{\sum }}F_{i},\\ F_{i}\approx b\omega_{i}^{2}, \end{array}$$

where *F*_*i*_ is the force generated by the rotor *i* and × means the vector product.

*F*_*t*_ is the drag force along the axes *x*, *y*, and *z*:

(7) $$\begin{array}{c} F_{t}=\left[\begin{array}{ccc} -K_{ftx} & 0 & 0\\ 0 & -K_{fty} & 0\\ 0 & 0 & -K_{ftz} \end{array}\right]v=\left[\begin{array}{c} -K_{ftx}\;\dot{x}\\ -K_{fty}\;\dot{y}\\ -K_{ftz}\;\dot{z} \end{array}\right], \end{array}$$

where *K*_*ftx*_, *K*_*fty*_, and *K*_*ftz*_ are the translational drag coefficients.

*F*_*g*_ is the gravity vector:

(8) $$\begin{array}{c} F_{g}=\left[\begin{array}{c} 0\\ 0\\ -mg \end{array}\right]. \end{array}$$

*M*_*f*_ is the moment caused by the thrust and the drag forces following the three rotations:

(9) $$\begin{array}{c} M_{f}=\left[\begin{array}{c} M_{fx}\\ M_{fy}\\ M_{fz} \end{array}\right]=\left[\begin{array}{c} lb\left(\omega_{4}^{2}-\omega_{2}^{2}\right)\\ lb\left(\omega_{3}^{2}-\omega_{1}^{2}\right)\\ d\left(\omega_{1}^{2}-\omega_{2}^{2}+\omega_{3}^{2}-\omega_{4}^{2}\right) \end{array}\right]. \end{array}$$

*M*_*a*_ is the moment resulting from aerodynamic friction:

(10) $$\begin{array}{c} M_{a}=\left[\begin{array}{c} K_{fax}{\dot{\phi }}^{2}\\ K_{fay}{\dot{\theta }}^{2}\\ K_{faz}{\dot{\psi }}^{2} \end{array}\right], \end{array}$$

where *K*_*fax*_, *K*_*fay*_, and *K*_*faz*_ are coefficients of aerodynamic friction.

*M*_*gh*_ is the gyroscopic moment of the propellers:

(11) $$\begin{array}{c} M_{gh}=\overset{4}{\underset{i=1}{\sum }}\Omega \times J_{r}\left[\begin{array}{ccc} 0 & 0 & {\left(-1\right)}^{i+1}\omega_{i} \end{array}\right]=\left[\begin{array}{c} J_{r}\Omega_{r}\dot{\theta }\\ -J_{r}\Omega_{r}\dot{\phi }\\ 0 \end{array}\right], \end{array}$$

where *J*_*r*_ is the inertia of the rotors and Ω_*r*_ *=* *ω*_1_ − *ω*_2_ *+* *ω*_3_ − *ω*_4_;

*M*_*gm*_ is the gyroscopic moment due to the movements of the quadrotor:

(12) $$\begin{array}{c} M_{gm}=\Omega \times J\Omega . \end{array}$$

Then, the dynamic model of the quadrotor can be expressed by the following equations, as given in [1]:

(13) $$\begin{array}{c} \ddot{\phi }=\frac{l\;U_{2}}{I_{x}}+\frac{\left(I_{y}-I_{z}\right)}{I_{x}}\left(\dot{\psi \;}\cos\;\phi \;\cos\;\theta -\dot{\theta }\sin\;\phi \right)\left(\dot{\theta }\cos\;\phi +\dot{\psi }\;\sin\;\phi \;\cos\;\theta \right)-\frac{J_{r}\left(\omega_{1}-\omega_{2}+\omega_{3}-\omega_{4}\right)}{I_{x}}\left(\dot{\psi \;}\sin\;\phi \;\cos\;\theta +\dot{\theta }\;\cos\;\phi \right)-\frac{K_{fax}}{I_{x}}\left({\dot{\phi }}^{2}-2\dot{\phi }\dot{\psi \;}\sin\;\theta^{2}\right), \end{array}$$

(14) $$\begin{array}{c} \ddot{\theta }=\frac{l\;U_{3}}{I_{y}}+\frac{\left(I_{z}-I_{x}\right)}{I_{y}}\left(\dot{\psi }\;\cos\;\phi \;\cos\;\theta -\dot{\theta \;}\sin\;\phi \right)\left(\dot{\phi }-\dot{\psi \;}\sin\;\theta \right)-\frac{J_{r}\left(\omega_{1}-\omega_{2}+\omega_{3}-\omega_{4}\right)}{I_{y}}\left(\dot{\psi \;}\sin\;\theta -\dot{\phi }\right)-\frac{K_{fay}}{I_{y}}\left({\dot{\theta }}^{2}\;\cos\;\phi^{2}+2\dot{\phi }\;\dot{\psi }\;\sin\;\phi \;\cos\;\phi \;\cos\;\theta \;+{\dot{\psi }}^{2}\;\sin\;\phi^{2}\;\cos\;\theta^{2}\right), \end{array}$$

(15) $$\begin{array}{c} \ddot{\psi }=\frac{U_{4}}{I_{z}}+\frac{\left(I_{x}-I_{y}\right)}{I_{z}}\left(\dot{\psi \;}\sin\;\phi \;\cos\;\theta +\dot{\theta }\;\cos\;\phi \right)\left(\dot{\phi }-\dot{\psi \;}\sin\;\theta \right)-\frac{J_{r}\left(\omega_{1}-\omega_{2}+\omega_{3}-\omega_{4}\right)}{I_{y}}\left(\dot{\psi }\;\sin\;\theta -\dot{\phi }\right)-\left({\dot{\theta }}^{2}\sin\;\phi^{2}-2\dot{\phi }\dot{\psi }\;\sin\;\phi \;\cos\;\phi \;\cos\;\theta \right)+\frac{K_{faz}}{I_{z}}{\dot{\psi }}^{2}\cos\;\phi^{2}\cos\;\theta^{2}, \end{array}$$

(16) $$\begin{array}{c} \ddot{x}=\frac{\cos\;\phi \;\cos\;\psi \;\sin\;\theta +\sin\;\phi \;\sin\;\psi }{m}U_{1}-\frac{K_{ftx}}{m}\dot{x}, \end{array}$$

(17) $$\begin{array}{c} \ddot{y}=\frac{\sin\;\psi \;\sin\;\theta \;\cos\;\phi -\cos\;\psi \;\sin\;\phi }{m}U_{1}-\frac{K_{fty}}{m}\dot{y}, \end{array}$$

(18) $$\begin{array}{c} \ddot{z}=-g+\frac{\cos\;\phi \;\cos\;\theta }{m}U_{1}-\frac{K_{ftz}}{m}\dot{z}, \end{array}$$

with

(19) $$\begin{array}{c} \left\{\begin{array}{c} U_{1}=b\left(\omega_{1}^{2}+\omega_{2}^{2}+\omega_{3}^{2}+\omega_{4}^{2}\right),\\ U_{2}=b\left(\omega_{4}^{2}-\omega_{2}^{2}\right),\\ U_{3}=b\left(\omega_{3}^{2}-\omega_{1}^{2}\right),\\ U_{4}=d\left(\omega_{1}^{2}-\omega_{2}^{2}+\omega_{3}^{2}-\omega_{4}^{2}\right). \end{array}\right. \end{array}$$

Table 1 shows the definitions of the quadrotor's parameters.

## 3. Identification Strategy

Simulations of the quadrotor dynamical model, expressed by equations (13)–(18), with a unit step reference signal for *U*_1_, *U*_2_, *U*_3_, and *U*_4_, give unstable responses. The instability observed for the four flight variables is of divergent type, which can be simply modelled by second-order systems. In addition, the flight parameters of the quadrotor can be separated, thus giving four subsystems of order 2 having the same form *G*_*mi*_ (*p*), with *i* = {*ϕ, θ, ψ, z*}.

(20) $$\begin{array}{c} G_{mi}\left(p\right)=\frac{K_{mi}}{\left(p+a_{mi}\right)\left(p+b_{mi}\right)}. \end{array}$$

**Figure 2:**
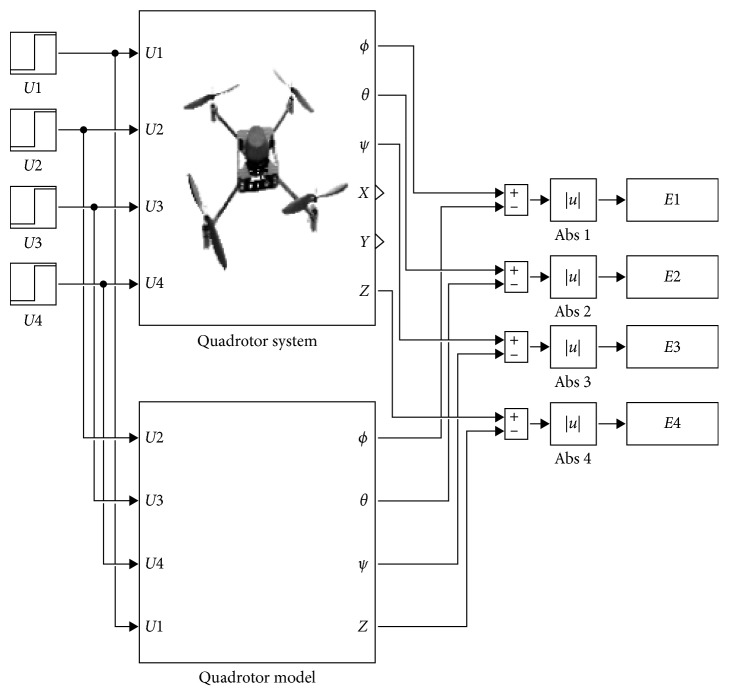
Quadrotor identification scheme.

When the model of system is fixed, the identification task can be treated as an optimization problem. The basic idea of parameter estimation is to compare the time-dependent responses of the system and the model based only on inputs and outputs data [5]. Considering Figure 2, *U*_*k*_ are the excitation inputs and *E*_*k*_ are the errors of identification that characterize the difference in behavior between the quadrotor system and model, with *k* = {1, 2, 3, 4}. Then, PSO, CS, PSO-CS, and LS are used as adequate methods to optimally estimate the quadrotor's model parameters (*K*_*mi*_, *a*_*mi*_, and *b*_*mi*_).

## 4. Quadrotor Identification Using Intelligent Methods

### 4.1. Intelligent PSO

For hard optimization problems, particle swarm optimization (PSO) was developed by Eberhart and Kennedy in 1995. The basic principle of PSO was inspired by the social behavior of animals moving in a swarm as bird flocking. To search for food, each bird flies in the space of solutions and determines its speed according to its personal experience and the information obtained through interaction with other swarm members [4].

The initialization matrix contains *N* particles dispersed in a search space along dimension *j* for *j* = {1, 2,…, *D*}. Each particle *P*_*i*_ (*i* *=* 1, 2,…, *N*) stores its best position *Pb*_*i*_ (*t* *+* 1) and the best solution in its vicinity *Pg* (*t* *+* 1), which is the position of the particle that has the smallest fitness value in the swarm as expressed in equation (22). The mechanism of displacement of each particle is managed by three rules. Firstly, the particle tends to follow the direction of its current velocity. Secondly, it tends to move towards its best position. Finally, it tends to move to the best position reached by its neighbors [5, 23]. In fact, the new velocity matrix *V*_*ij*_ *a*nd position matrix *X*_*ij*_ of particles are calculated at iteration (*t* *+* 1), according to equations (23) and (24):

(21) $$\begin{array}{c} Pb_{i}\left(t+1\right)=\left\{\begin{array}{c} X_{i}\left(t+1\right),\;\text{if }f\left(X_{i}\left(t+1\right)\right)<f\left(Pb_{i}\left(t\right)\right),\\ Pb_{i}\left(t\right),\;\text{else,} \end{array}\right. \end{array}$$

(22) $$\begin{array}{c} P_{g}=\underset{i=1,2,\ldots ,N}{\min}f\left(Pb_{i}\left(t\right)\right), \end{array}$$

(23) $$\begin{array}{c} V_{ij}\left(t+1\right)=w\cdot V_{ij}\left(t\right)+R_{1}C_{1}\;⊗\;\left(Pb_{ij}\left(t\right)-X_{ij}\left(t\right)\right)+R_{2}C_{2}\;⊗\;\left(Pg_{ij}\left(t\right)-X_{ij}\left(t\right)\right), \end{array}$$

(24) $$\begin{array}{c} X_{ij}\left(t+1\right)=X_{ij}\left(t\right)+V_{ij}\left(t+1\right), \end{array}$$

where *Pb*_*ij*_ is the best position found by the particle *i*; *Pg*_*ij *_Pbest_*ij*_(*g* − 1) is the best position found by the neighborhood; *w*, *C*_1_, and *C*_2_ are weighting coefficients; and *R*_1_ and *R*_2_ are random variables generated from a uniform distribution in [0, 1].

### 4.2. Intelligent CS

Cuckoo search (CS) algorithm, proposed by Yang and Deb in 2009, is based on the life of a “cuckoo” bird. The basic principle of CS is the specific breeding and egg laying of this bird. In the habitat of other host birds, adult cuckoos lay some eggs that grow and become mature cuckoos if are not discovered and removed by host birds. Reproduction and breeding are favoured by cuckoo groups' immigration, converging and reaching the best places [10].

The primary population of CS contains *N* nests, and each nest is composed of *D* eggs. The best nests with a high quality of eggs (solutions) carry over to the next generations, where the quality evaluation is based on the fitness function *F* of the habitat (array of 1 × *D*). The host can discover an alien egg with probability *P*_*a*_ from [0, 1], which is approximated by a fraction *P*_*a*_ of the *N* nests being replaced by new nests, having new random solutions [24].

To explore the search space when replacing solutions in the nests with new solutions, *Lévy* flight mechanism is used. The step length *S* from Mantegna algorithm (based on Gaussian normal distribution denoted by Norm) can be written as represented by equation (25), and *σ*_*u*_^*2*^ is the variance of the distributions given by equation (26). Therefore, a new solution *X*_*i*_ (*t* *+* 1) for cuckoo *i* is given by equation (27), and the fraction *P*_*a*_ of worse solutions is generated as given by equation (28):

(25) $$\begin{array}{c} L\text{é}vy\left(\lambda \right)\approx S=\left(\text{Norm}\left(0,\sigma_{u}^{2}\right)\right){\left|\text{Norm}\left(0,1\right)\right|}^{-1/\lambda },\;\left(1<\lambda \leq 3\right), \end{array}$$

(26) $$\begin{array}{c} \sigma_{u}^{2}={\left\{\frac{\Gamma \left(1+\lambda \right)\sin\left(\pi \lambda /2\right)}{\Gamma \left[\left(1+\lambda \right)/2\right]\lambda 2^{\left(\lambda -1\right)/2}}\right\}}^{1/\lambda }, \end{array}$$

(27) $$\begin{array}{c} X_{i}\left(t+1\right)=X_{i}\left(t\right)+\alpha \;⊗\;L\text{é}vy\left(\lambda \right), \end{array}$$

(28) $$\begin{array}{c} X_{i}\left(t+1\right)=X_{i}\left(t\right)+\left(P_{a}-r\right)\left(X_{j}\left(t\right)-X_{k}\left(t\right)\right), \end{array}$$

where Γ is the gamma function; *α* > 0 is the step size; *R* and *r* are random variable generated from a uniform distribution in the interval [0, 1]; *X*_*j*_ (*t*) and *X*_*k*_ (*t*) are two random solutions chosen by random permutation; and *H* is a Heaviside function.

### 4.3. Cooperative PSO-CS

The initialization matrix of the cooperative PSO-CS is of dimension *D* × *N*, and the solutions' quality is evaluated as in PSO and CS. The global best particle (or the best nest) is the particle (or the nest) that has the smallest fitness value among all potential solutions [22].

To overcome the fast convergence speed of PSO and the low convergence speed of CS, the PSO-CS combines the capacities of social thinking in PSO and local search in CS. Thus, the displacement equation is modified, by combining the *Lévy* flight random walks of cuckoos and the velocity of particles toward the global best solution *Pg*. New solutions *X*_*i*_ (*t* *+* 1) are given by [22]

(29) $$\begin{array}{c} X_{i}\left(t+1\right)=X_{i}\left(t\right)+\alpha \;⊗\;L\text{é}vy\left(\lambda \right)+R_{2}C_{2}\;⊗\;\left(Pg\left(t\right)-X_{i}\left(t\right)\right). \end{array}$$

**Figure 3:**
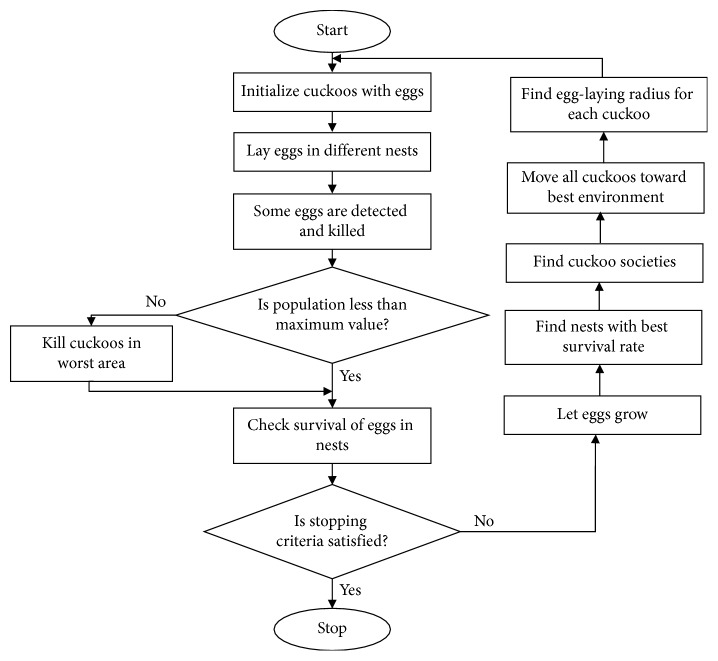
Flow chart of CS and PSO-CS.

Both algorithms' capabilities are combined to increase the particles' diversification. PSO-CS guides the cuckoos toward the global best positions (global intelligence of the swarm). In fact, the search ability increases during iterations, and the exploration of the local and global places is achieved by the Lévy flight displacement of cuckoos and the velocity of particles toward the global best solution (*Pg*), as given by equation (29). The flow chart of PSO-CS is considered the same as that of CS. It is given in Figure 3. The only modification is in the expression of the velocity of displacement, which helps to search at local and global scales in order to move all cuckoos toward best environment and to quickly converge at later stage.

### 4.4. Parameter Setting for PSO, CS, and PSO-CS

Intelligent PSO, CS, and PSO-CS are applied to optimally select the model parameters from *N* = 200 solutions. So, the search space interval is chosen sufficient to contain all possible solutions [0, 200], and its dimension *D* is set to 12. The compromise between local and global exploration in PSO is achieved for *w* = 0.8. *C*_1_ takes random values in [0, 0.8] to avoid the problem of fast convergence, while *C*_2_ takes random values in [0, 1.2] to give more importance to the global best solution *Pg* [22]. While in CS, the parameters used in experiments are as follows: abandon probability, *P*_*a*_ = 0.25, and the Lévy flight settings, *α* = 0.1 and *λ* = 1.5 [22]. In PSO-CS, the same settings of PSO and CS are kept [22]: *P*_*a*_ = 0.25 and the Lévy flight settings *α* = 0.1, *λ* = 1.5, and *C*_2_ with random values in [0, 1.2].

These algorithms are evaluated using a profit defined in a similar way in order to minimize the differences between the output responses of the estimated model and the quadrotor system. This fitness function is defined in equation (30) as the sum of the quadratic errors *E*_*k*_ previously mentioned, with *l* = 4 and *k* = {1, 2, 3, 4}:

(30) $$\begin{array}{c} F=\frac{1}{2}\overset{l}{\underset{k=1}{\sum }}E_{k}^{2}. \end{array}$$

An appropriate set of PSO, CS, and PSO-CS parameters can yield model responses close to those of the quadrotor. The maximum number of generations for the three programs (PSO, CS, and PSO-CS) is fixed as the stop criterion and set to 20.

## 5. Quadrotor Identification Using the Statistical LS Method

The method of least squares (LS) provides the parameters of a model so that the sum of squared errors (between the predicted and observed values) is minimal [25].

For the quadrotor's identification, we consider the same identification scheme represented in Figure 2. The vectors of measurements are extracted from the temporal responses of the quadrotor's attitude (*ϕ, θ, ψ*) and altitude *z*.

The recurring equation in equation (31) is obtained by discretizing at *T*_*e*_ the temporal response of each transfer function *G*_*mi*_ (*p*) (in equation (20)), where *u*_*k*_ and *y*_*k*_ are, respectively, discrete inputs and outputs of *G*_*mi*_ (*p*):

(31) $$\begin{array}{c} y\;\left(k+2\right)=-a_{0}y\left(k\right)-\;a_{1}\;y\left(k+1\right)+\;b_{0}u\left(k\right)+b_{1}u\left(k+1\right). \end{array}$$

The *a*_*j*_ and *b*_*k*_ coefficients or *θ* (equation (32)) to be estimated, in the sense of the LS criterion given in equation (33), is computed following equation (34). The matrix *F* and the vector *Y* are constituted from the values taken by *u* and *y* at the different sampling times:

(32) $$\begin{array}{c} \hat{\theta }=\left(\begin{array}{c} a_{0}\\ \begin{array}{c} a_{1}\\ b_{0}\\ b_{1} \end{array} \end{array}\right), \end{array}$$

(33) $$\begin{array}{c} J\;\left(\hat{\theta }\;\right)=\frac{1}{2}\sum {\left(\varepsilon \left(i\right)\right)}^{2}, \end{array}$$

(34) $$\begin{array}{c} \hat{\theta }={\left(F^{T}F\right)}^{-1}\left(F^{T}Y\right), \end{array}$$

(35) $$\begin{array}{c} F=\left(\begin{array}{cccc} -y_{0}\; & -y_{1} & u_{0} & u_{1}\\ -y_{1} & -y_{2} & u_{1} & u_{2}\\ \; & \vdots & \; & \;\\ -y_{N-2} & -y_{N-1} & u_{N-2} & u_{N-1} \end{array}\right), \end{array}$$

The transition between continuous (*k*_*m*_ *, a*_*m*_ *, b*_*m*_) and discrete parameters (*a*_0_ *, a*_1_, *b*_0_ *b*_1_) is grouped in the following equations:

(36) $$\begin{array}{c} \left\{\begin{array}{c} a_{0}=-e^{-\left(a_{m}+b_{m}\right)T_{e}},\\ a_{1}=-e^{-b_{m}T_{e}}-e^{-a_{m}T_{e}},\\ b_{0}=\frac{K_{m}}{a_{m}b_{m}}\left[e^{-\left(a_{m}+b_{m}\right)T_{e}}+\frac{b_{m}}{a_{m}-b_{m}}e^{-b_{m}T_{e}}+\frac{a_{m}}{a_{m}-b_{m}}e^{-a_{m}T_{e}}+1\right]\;,\\ b_{1}=\frac{K_{m}}{a_{m}b_{m}}\left[-e^{-b_{m}T_{e}}-e^{-a_{m}T_{e}}-\frac{b_{m}}{a_{m}-b_{m}}e^{-b_{m}T_{e}}-\frac{a_{m}}{a_{m}-b_{m}}e^{-a_{m}T_{e}}\right], \end{array}\right. \end{array}$$

(37) $$\begin{array}{c} \left\{\begin{array}{c} K_{mi}=\;\frac{a_{mi}b_{mi}\left(b_{0}+b_{1}\right)}{1+a_{0}+a_{1}},\\ a_{mi}=-\frac{1}{T_{e}}\log\left(\frac{-a_{0}+\sqrt{a_{0}^{2}²\;-\;a_{1}}}{2}\right),\\ b_{mi}=-\frac{1}{T_{e}}\log\left(a_{1}\right)-a_{mi}. \end{array}\right. \end{array}$$

The application of this approach to the identification of the quadrotor's movements is discussed in the following section.

## 6. Simulation Results

**Figure 4:**
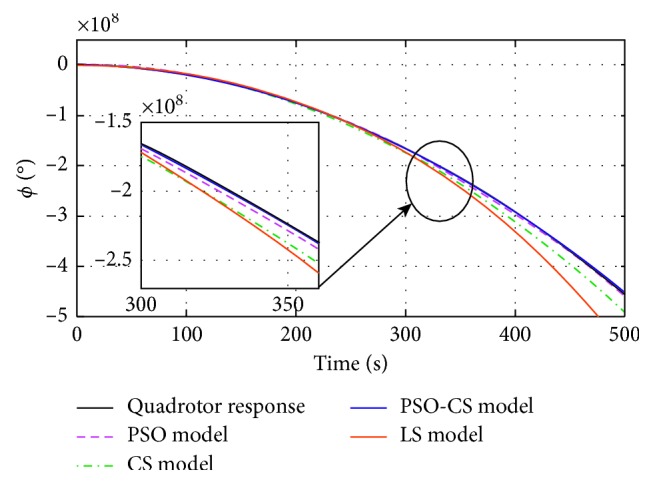
Roll (*ϕ*) responses of the quadrotor and the model *G*_*mϕ*_(*p*), obtained using PSO, CS, PSO-CS, and LS.

**Figure 5 fig5:**
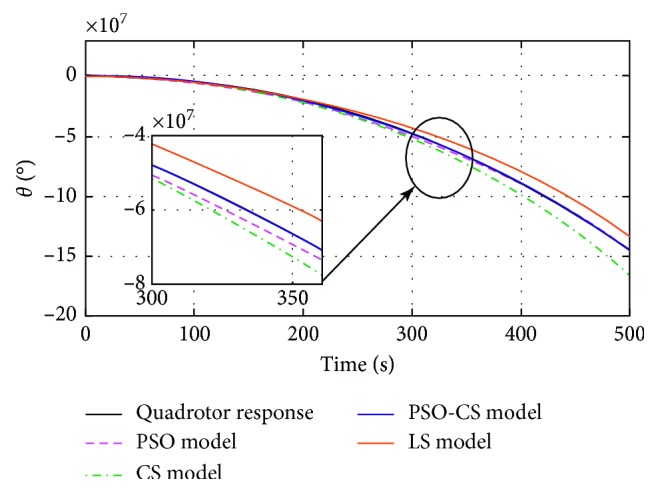
Pitch (*θ*) responses of the quadrotor and the model *G*_*mθ*_(*p*), obtained using PSO, CS, PSO-CS, and LS.

**Figure 6 fig6:**
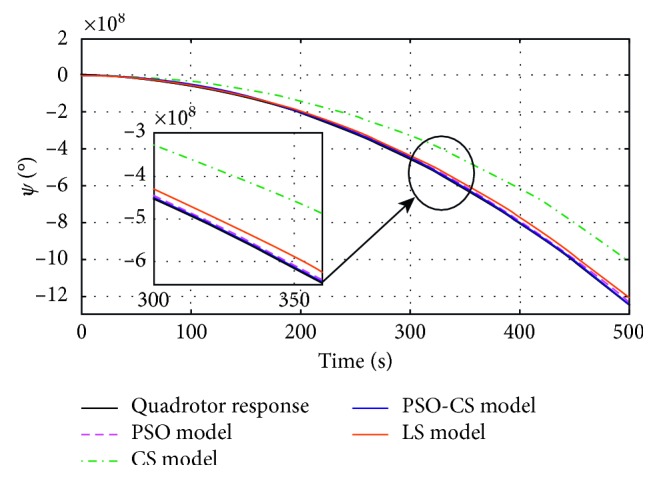
Yaw (*ψ*) responses of the quadrotor and the model *G*_*mѱ*_(*p*), obtained using PSO, CS, PSO-CS, and LS.

**Figure 7:**
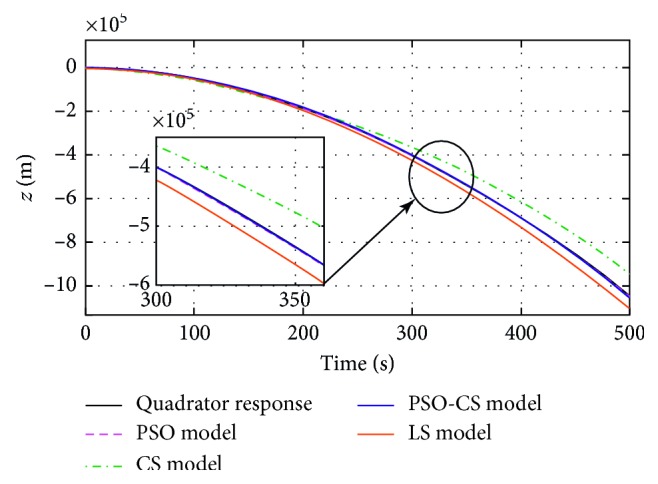
Altitude (*z*) responses of the quadrotor and the model *G*_*mz*_(*p*), obtained using PSO, CS, PSO-CS, and LS.

The quadrotor system is identified using a model composed of four subsystems with the same structure, expressed by *G*_*mi*_ (*p*) in equation (20). The quadrotor model is generated in a simulation time of 500 seconds.  Table 2 summarizes the parameters of these subsystems (*K*_*mi*_ *, a*_*mi*_ *, b*_*mi*_) estimated by using PSO, CS, PSO-CS, and LS methods, with *i* *=* {*ϕ, θ, ψ, z*}. Figures 4 [Fig fig5] [Fig fig6]–7 show the responses of the quadrotor's system and models with parameters obtained using PSO, CS, PSO-CS, and LS, in an observation time of 500 seconds.  Table 3 shows the results of statistical analysis based on the integral of a positive term involving the error: the integral absolute error (IAE), the integral square error (ISE), the integral time absolute error (ITAE), and the integral time square error (ITSE) [6].

PSO and PSO-CS perform the search with high precision to avoid all solutions that are far from the desired responses of the quadrotor system (angles of roll, pitch, yaw, and altitude *z*). From Figures 4 [Fig fig5] [Fig fig6]–7, the model responses with parameters adjusted using PSO and PSO-CS are very close to those of the quadrotor's responses, while CS and LS give the estimated model parameters that allow fitting a divergent behavior near to that of the quadrotor system.

These observations spread to 500 seconds prove the validity of the chosen model to identify the divergent responses of the quadrotor, as well as the efficiency of PSO and PSO-CS for seeking for the best solution, even if identifying unstable responses is a very difficult task. During the simulation time, PSO and PSO-CS effectively avoid the divergence that can appear between the model (20) with the calculated parameters and the quadrotor system defined by equations (13)–(18). PSO and PSO-CS reduce the fitness function and result in an optimal search for the parameters of the models. To confirm the effectiveness of PSO and PSO-CS in reducing the errors between the two responses for the four outputs, we present in the following table a statistical analysis based on the integral of the errors IAE, ISE, ITAE, and ITSE.

Statistical results in Table 3 show that the errors between the model outputs and the system outputs, such as IAE, ISE, ITAE, and ITSE are all smaller (higher performance) when the model is established by PSO-CS than PSO, CS, and LS.

In [26], the authors proposed a modified CS algorithm named oriented cuckoo search algorithm (OCS), where they have tested its performance for different probability distributions (DDICS1, DDICS2, MCS1, MCS2, DACS1, DACS2, and OCS-LG). The proposed oriented cuckoo search algorithm with Lévy distribution and standard Gaussian distribution (OCS-LG) has shown better performance in means of mean error. The lower value means the better performance; therefore, OCS-LG is superior to other six algorithms. From the results of Wilcoxon test, there are significant differences (*p* value below 0.05) among OCS-LG, DACS1, MCS1, and MCS2. In other words, OCS-LG is significantly better than DACS1, MCS1, and MCS2.

The proposed hybrid many-objective cuckoo search (HMaOCS) for many-objective optimization problems (MaOPs) in [27] show that HMaOCS is promising in dealing with most many-objective optimization problems. It was noted that in CS, there is no guidance information (the global best individual) and each individual in the population is not affected by any other individuals. Then, the proposed changes were focused in the population updating method, where the new population was generated from the combined parent and offspring populations to ensure better convergence and diversity. However, the computation time necessary to generate new solutions in this way was not considered.

Comparisons to OCS-LG and HMaOPs show that our proposed PSO-CS algorithm used *Lévy* flight mechanism with Gaussian normal distribution when replacing solutions in the nests with new solutions, which is the best solution attested in [26]. In addition, PSO-CS benefits from the global intelligence already calculated in each iteration in order to manage the displacements of solutions.

Our proposed programs are performed on a computer with a processor Intel® Core™ i7-3770 CPU of 3.40 GHz and 8.0 GB of RAM using Matlab R2016a. The implemented PSO-CS program consumes an additional CPU time (6 min 15 s) than PSO and CS programs that spend (5 min 07 s) and (5 min 50 s), respectively. This additional simulation time of PSO-CS is produced due to the sum of both matrices generating Lévy flight random walks of cuckoos (*X*_*i*_(*t*) + *α*  ⊗  *L*é*vy*(*λ*))  and the movement of particles toward the global best solution *R*_2_ *C*_2_  ⊗  (*Pg*(*t*) − *X*_*i*_(*t*)). This updating method is faster than generating solutions from the combined parent using selection and crossover operators.

## 7. Conclusions

In this paper, recent heuristics optimization of particle swarm optimization (PSO) and cuckoo search (CS), the proposed cooperative particle swarm optimization-cuckoo search (PSO-CS), and the statistical least squares (LS) has been applied to identify the variations of the rotational movements (*ϕ*, *θ*, and *ψ* angles) and the translational movement along *z*-axis of a quadrotor UAV. Simulation results proved the efficiency of the PSO approach and the cooperative PSO-CS in identifying optimally the quadrotor's outputs (*ϕ*, *θ*, *ψ*, *z*) compared to CS and LS methods. The proposed PSO-CS seeks for the best solution by exploiting the local search capacity of CS and benefiting from the global intelligence offered by PSO.

These achievements are the results of the good choice of the model structure to identify the quadrotor system responses with unstable behaviors and the good adjustment of PSO, CS, and PSO-CS parameters (the fitness function, the weighting coefficients *w*, *C*_1_, and *C*_2_, the probability *P*_*a*_, and the *Lévy* flight settings for *α* and *λ*). However, PSO-CS program consumes an additional CPU time to calculate the velocity update equation, and a wrong setting of the acceleration constant *C*_2_ can cause premature convergence.

## Figures and Tables

**Table 1:** The parameters of the quadrotor.

Parameter	Definition	Value
*I*_*x*_ (kg·m^2^)	The inertia moments with respect to the axes	7.5 · 10^−3^
*I*_*y*_ (kg·m^2^)		7.5 · 10^−3^
*I*_*z*_ (kg·m^2^ )		1.3 · 10^−3^

*K*_*fax*_ (N/rad/s)	The aerodynamic coefficients	5.567 · 10^−4^
*K*_*fay*_ (N/rad/s)		5.567 · 10^−4^
*K*_*faz*_ (N/rad/s)		6.354 · 10^−4^

*K*_*ftx*_ (N/rad/s)	The drag coefficients	5.567 · 10^−4^
*K*_*fty*_ (N/rad/s)		5.567 · 10^−4^
*K*_*ftz*_ (N/rad/s)		6.354 · 10^−4^

*g* (m/s)	The gravity acceleration	9.806
*J*_*r* _ (kg·m^2^)	The inertia of the rotors	2.8385 · 10^−5^
*b *(kg·m·rad^−2^)	The thrust coefficient	2.9842 · 10^−5^
*d* (kg·m·rad^−2^)	The drag coefficient	3.232 · 10^−6^
*m* (kg)	The total mass of the quadrotor	0.65
*l* (m)	The half span of the quadrotor	0.23

**Table 2:** Model's parameters given by PSO, CS, PSO-CS, and LS methods.

Optimization method	Parameter	*G*_*mф*_ (*p*)	*G*_*mθ*_ (*p*)	*G*_*mψ*_ (*p*)	*G*_*mz*_ (*p*)
PSO	*K*_*mi*_	−3990.4	−1093.62	−9890.68	−9.95
	*a*_*mi*_	−0.0009	0.0002	0.0001	−0.0001
	*b*_*mi*_	0.0016	−0.0005	−0.0001	0.0012

CS	*K*_*mi*_	−3850.82	−940.99	−6240	−15.589
	*a*_*mi*_	−0.001	−0.002	−0.0016	−0.003
	*b*_*mi*_	0.001	0.0001	1.260 · 10^−4^	0.015

PSO-CS	*K*_*mi*_	−3880	−935.561	−9973.1	−9.6921
	*a*_*mi*_	0.0009	−0.0011	−2.812 · 10^−4^	−8.745 · 10^−4^
	*b*_*mi*_	−0.0004	1.767 · 10^−4^	2.845 · 10^−4^	0.0019

LS	*K*_*mi*_	−3336	−908.74	−9447.8	−10.31
	*a*_*mi*_	−0.0033	−0.0032	−8.218 · 10^−4^	−2.639 · 10^−5^
	*b*_*mi*_	0.0026	0.0037	7.657 · 10^−4^	0.0010

**Table 3:** Statistical analysis of predicted errors using PSO, CS, and PSO-CS models.

Predictive output		IAE	ISE	ITAE	ITSE
*ϕ*	PSO model	2.3482	0.8960	8.3712	34.6600
	CS model	9.4593	2.0600	38.0080	899.4400
	PSO-CS model	0.6122	0.1318	2.4165	6.1527
	LS model	18.775	11.127	79.059	5090.6

*θ*	PSO model	1.8351	7.75 · 10^−2^	7.2906	33.9510
	CS model	5.0279	0.5808	19.9930	256.4700
	PSO-CS model	1.6507	0.0389	4.6952	11.1720
	LS model	4.6466	0.4001	17.590	165.32

*ψ*	PSO model	3.6325	0.2550	13.9370	107.6700
	CS model	99.0120	155.2600	355.9000	61671
	PSO-CS model	0.1661	3.36 · 10^−2^	0.4550	9.87 · 10^−2^
	LS model	16.216	4.3374	59.174	1748

*z*	PSO model	2.6 · 10^−3^	2 · 10^−7^	1.04 · 10^−2^	9.2040 · 10^−5^
	CS model	3.56 · 10^−2^	2.51 · 10^−5^	0.1363	1.0552 · 10^−2^
	PSO-CS model	1 · 10^−3^	1 · 10^−8^	2.8 · 10^−3^	4.15 · 10^−6^
	LS model	1.93 · 10^−2^	6.58 · 10^−6^	7.195 · 10^−2^	2.7307 · 10^−3^

## Data Availability

No data were used to support this study.
